# Mapping Progress in Workplace Protections: A Study of Global LGBTQI + Workplace Antidiscrimination Legislation in 2016 and 2023

**DOI:** 10.1007/s13178-024-01074-4

**Published:** 2025-01-30

**Authors:** Ross L. Weistroffer, Amy Raub, Aleta Sprague, Jody Heymann

**Affiliations:** 1 Independent Researcher, Los Angeles, CA USA; 2 WORLD Policy Analysis Center, University of California, Los Angeles, Los Angeles, CA USA

**Keywords:** LGBTQI +, Antidiscrimination, Discrimination, Workplace, Employment, Labor, Comparative policy analysis

## Abstract

**Introduction:**

Despite global commitments to advance economic inclusion for all, lesbian, gay, bisexual, transgender, queer, and intersex (LGBTQI +) workers around the world continue to face workplace discrimination based on their sexual orientation, gender identity, gender expression, and sex characteristics (SOGIESC). Developing a detailed global understanding of national laws addressing SOGIESC-based employment discrimination is a critical step towards making progress in these commitments.

**Methods:**

In this study, we systematically analyzed how detailed measures of legal prohibitions of SOGIESC-based employment discrimination across hiring, pay, promotions, access to training, and terminations changed from 2016 to 2023. Additionally, we examined measures that support implementation and enforcement, as well as exceptions to protections. We also assessed differences in protections between regions as classified by the World Bank using Pearson’s Chi-squared tests.

**Results:**

We found that 71 countries prohibited at least some form of workplace discrimination based on sexual orientation, 41 countries did so based on gender identity, 18 did so based on gender expression, and 14 did so based on sex characteristics.

**Conclusions:**

Notable legal gaps remained in prohibition specificity, nationwide coverage, and enforcement mechanisms. Protections are also undermined by exemptions for faith-based institutions found in 4 countries. While progress in protections is sizable and steady, inequities persist across regions.

**Policy Implications:**

These findings offer actionable insights regarding the strengths and shortcomings of current national laws and can act as the foundation for further legal progress in protecting LGBTQI + people at work.

## Introduction

### Background

Worldwide, lesbian, gay, bisexual, transgender, queer, and intersex (LGBTQI +) people face discrimination at work on the basis of their sexual orientation, gender identity, gender expression, and sexual characteristics (SOGIESC), threatening their livelihoods, their well-being, and potentially their lives. Beyond its direct impacts on employment outcomes, workplace discrimination is a documented lifetime risk factor for suicidal thoughts and behaviors among transgender and gender diverse adults (Cramer et al., 2022), and the stress produced by discrimination at work directly impacts LGBTQI + workers’ mental and physical well-being (Moya & Moya-Garófano, 2020). While the Global Attitudes Survey reports double-digit increases in rates of reported public acceptance of homosexuality over the past two decades in many countries around the world (Poushter & Kent, 2020) discrimination at work remains widespread even in countries with high rates of public acceptance of LGBTQI + people (Drydakis, 2021b; Sears et al., 2021). Laws and policies prohibiting discrimination at work are important for providing legal recourse when discrimination occurs and are strongly correlated with greater societal acceptance of LGBT people (Flores & Park, 2018). This study provides the first in-depth look at whether legislation specifically prohibits SOGIESC discrimination at work, as well as whether there are provisions in place to support the effective implementation of these laws, across 193 countries.

### SOGIESC-Based Discrimination Across the Employment Experience

A wide array of research has documented how LGBTQI + workers face discrimination across different aspects of the employment experience. Although the majority of this research has focused on high-income countries, its topical diversity illustrates the multifaceted and systemic nature of the barriers LGBTQI + people face in access to meaningful work. Discrimination in hiring is particularly well-documented by correspondence studies, which typically compare how employers respond to fictitious job candidates whose CVs have equivalent qualifications, but differ in ways that would suggest they belong to a marginalized group. These studies find substantially lower chances that a job applicant whose CV implies they may be a gay or lesbian person (e.g., through membership in a gay campus organization) would receive an interview invitation in the UK (Drydakis, 2015), the U.S. (Tilcsik, 2011), Canada (Dilmaghani & Robinson, 2022), Greece (Drydakis, 2011), or the labor market of the Organization for Economic Co-operation and Development (OECD) countries more broadly (Drydakis, 2021b; Flage, 2020; Valfort, 2017). Field experiments focusing on transgender applicants find similar rates of hiring discrimination across European countries (Drydakis, 2021b; Granberg et al., 2020), while surveys of transgender workers reveal high rates of hiring discrimination on the basis of gender identity in Brazil (Costa et al., 2020), Colombia (Pérez Álvarez et al., 2013), Europe (European Union Agency for Fundamental Rights, 2015), and the USA (James et al., 2016). Studies indicate that SOGIESC-based hiring discrimination is especially concentrated in low-skilled (Flage, 2020) and blue-collar jobs (Dilmaghani & Robinson, 2022). As a result, LGBTQI + people of lower socioeconomic status, or who have faced discrimination in access to education or skill acquisition, may face even greater discriminatory barriers than average statistics imply.

Research has also documented significant wage discrimination based on sexual orientation, though findings vary across sub-groups. Recent meta-analyses find persistent earning penalties for gay men, bisexual men, and, to a lesser extent, bisexual women. By contrast, lesbian women often experience earning premiums (Drydakis, 2022; Ozeren, 2014; Weichselbaumer, 2022). Potential reasons for this variation include the gendered division of household responsibilities impacting the wages and work hours of heterosexual women (Wang & Gunderson, 2019) and the gender norms and stereotypes driving occupational segregation and related wage disparities (Pichler et al., 2010). Other studies conducted in the UK and Greece, though, still found that a wage penalty affects lesbian women (Bryson, 2017; Drydakis & Zimmermann, 2020). Such research suggests context-specific effects still produce notable discriminatory outcomes in pay for lesbian women relative to heterosexual women.

The consequences of wage discrimination on gay and bisexual men are clear. For example, despite controlling for job type and demographic characteristics, a wage gap of 30% persists for gender-nonconforming heterosexuals and gay and bisexual men in South Africa compared to their gender-conforming heterosexual counterparts (Nyeck et al., 2019). In the USA, wage penalties for gay men directly correlate with levels of prejudice (Burn, 2020), and in the UK, gay men are significantly less likely to be promoted to higher-level managerial positions that come with higher pay (Aksoy et al., 2019).

Surveys from a range of countries suggest that LGBTQI + workers are also disproportionately impacted by workplace harassment. For example, in the UK, 68% of LGBT workers surveyed reported being sexually harassed, while 21% of LGBT women surveyed reported being sexually assaulted (TUC, 2019). Fifteen percent of respondents to the U.S. Transgender Survey reported being verbally harassed, physically attacked, or sexually assaulted at their workplace within the past year because of their gender identity or expression (James et al., 2016). These and other discriminatory workplace pressures contribute to lower rates of openness about one’s LGBTQI + status at work, with only 50% of LGBTQI + workers choosing to disclose their gender identity or sexuality at work in the USA, dropping to 23% in the EU and 5% in China (Suen et al., 2021). Moreover, one US-based study found that respondents who were openly LGBTQI + at work were five times as likely to report experiencing discrimination at work on the basis of SOGIESC (Sears et al., 2021). However, hiding identities can take a devastating toll: a separate US study found that transgender workers who took steps to avoid such discrimination by concealing their gender identity or leaving their job were more likely to report suicidal thoughts and attempts (Herman et al., 2019).

### The Costs of Discrimination and the Benefits of Strong Antidiscrimination Laws

Discriminatory workplace conditions hinder LGBTQI + people’s access to labor markets and thereby their socioeconomic mobility. An econometric analysis of the French labor market finds uniquely higher unemployment risks and a significantly lower labor supply for gay workers (Laurent & Mihoubi, 2017), and similar findings note lower labor force participation rates by gay men in Chile and Uruguay (Brown et al., 2019). Transgender individuals are also affected: 14% of surveyed trans people in the USA report no income (Suárez et al., 2020); the number rises in Brazil to rates as high as 34% of surveyed transgender women and 45% of surveyed transgender men (Costa et al., 2020). Transgender women in the USA making below $50,000 a year and/or who have not completed university education are also significantly more likely to experience discriminatory outcomes at work than their higher-income and more educated counterparts (Suárez et al., 2020). In South Africa, gender nonconforming individuals are substantially less likely to be employed, especially if they are lesbian, gay, or bisexual, with the resulting economic costs adding up to an estimated US$316.8 million per year (Nyeck et al., 2019). LGBTQI + workers may also be more greatly impacted by economic downturns due to discrimination (Drydakis, 2021a).

Laws prohibiting discrimination are a first step towards addressing such inequalities and have effectuated substantial change in labor market discrimination on the basis of race and sex, among other grounds (Collins, 2003; Donohue & Heckman, 1991; Gunderson, 1989; Wright, 2015). Antidiscrimination workplace policies and legislation have direct causal effects on reducing workplace discrimination in hiring for LGBT workers (Barron & Hebl, 2013), reducing wage differentials for gay men (Burn, 2018; Martell, 2013; Preston et al., 2020) while increasing job satisfaction for gay and lesbian employees (Day & Schoenrade, 2000), and have been associated with lower exposure to discriminatory employment outcomes among transgender individuals (Suárez et al., 2020). Legal antidiscrimination protections are also associated with better health outcomes for LGBT people (Solazzo et al., 2018), better birth outcomes for lesbian and bisexual women (Everett et al., 2022), and significantly lower levels of self-reported internalized homophobia (Riggle et al., 2010).

These positive effects translate into economic benefits for firms and national economies. Antidiscrimination laws increase the labor supply and hours worked for gay men (Delhommer, 2020; Martell, 2014) and are also associated with increased national real GDP per capita (Badgett et al., 2019) and greater national innovative capacity (Vu, 2022). LGBT-supportive policies on the corporate level alone demonstrate increases in firm profitability (Pichler et al., 2018) and at least some limited protection against discriminatory outcomes for transgender individuals (Suárez et al., 2020). Moreover, antidiscrimination employment laws have the potential to improve attitudes towards LGBTQI + people on a societal scale, as studies have documented following the decriminalization of same-sex relationships (Kenny & Patel, 2017) and the passage of marriage equality laws (Flores & Barclay, 2016). Together, attitudes and legal protections covering economic activities can influence each other, potentially leading to virtuous cycles of “policy feedback” that forge a path towards greater societal acceptance of LGBTQI + people (Flores & Park, 2018).

### Addressing SOGIESC-based Discrimination at Work: International Foundations and Comparative Perspectives

Countries worldwide have made commitments to eliminate SOGIESC-based discrimination. For example, 171 countries have ratified, and an additional four have signed the International Covenant on Economic, Social, and Cultural Rights. In a 2016 General Comment—official guidance on how to interpret the Covenant’s provisions—the United Nations’ (UN) Committee on Economic, Social, and Cultural Rights made clear that the Convention’s articles on work, non-discrimination, and sexual health require states to prohibit workplace discrimination based on “sexual orientation, gender identity or intersex status” (2016). The General Comment built on repeated affirmations from other UN bodies that broad antidiscrimination provisions in international treaties should be interpreted to cover sexual orientation, gender identity, and sex characteristics (Office of the United Nations High Commissioner for Human Rights, 2019). Moreover, in 2015, every country around the world adopted the Sustainable Development Goals, through which they committed to advancing economic inclusion for all and “eliminat[ing] discriminatory policies and practices.”

An important step toward making progress on these commitments is understanding the state of national laws and policies addressing SOGIESC-based discrimination at work around the world. Mapping and monitoring the adoption of laws addressing employment is particularly valuable because prior research has not tracked change in this area in depth on a global scale. Prior globally comparative studies have examined constitutional rights across sexual orientation and gender identity or expression (Raub et al., 2016) and inequalities for same-sex couples embedded within paid parental leave policies (Wong et al., 2020). The same level of detailed analysis has not been available for employment discrimination laws. Both researchers and civil society organizations have previously compiled comprehensive information on the global recognition of same-sex rights, including the criminalization of same-sex conduct, same-sex relationship recognition, and adoption rights for same-sex couples, as well as taking an initial look at protections against employment discrimination (ILGA World et al., 2020; Waaldijk, 2009). However, the latter has been limited to a binary determination of whether discrimination in employment is prohibited based on sexual orientation.

This is the first global study to systematically analyze detailed measures of legal protections against employment discrimination based on sexual orientation, gender identity, gender expression, and sex characteristics across hiring, pay, promotions, access to training, and terminations, as well as legal requirements regarding implementation and prevention. In addition, this paper provides a comprehensive global overview of legal exceptions to workplace protections for LGBTQI + people, aiming to better understand potential weaknesses of different countries’ antidiscrimination laws. Finally, this study contributes to our understanding of global progress in the adoption of policies addressing SOGIESC-based discrimination at work by measuring how policies across all 193 countries have changed from 2016 to 2023. As the global community seeks to advance the realization of the International Covenant on Economic, Social, and Cultural Rights and the Sustainable Development Goals, alongside a range of other international instruments that guarantee equal rights to LGBTQI + workers, this study offers valuable and actionable insights about the strengths and shortcomings of current national laws.

## Methods

### Data Source

To assess the state of SOGIESC-relevant antidiscrimination policies around the world, we created and analyzed a legislative database of national-level laws covering the private sector from all 193 UN member states. Laws were assessed by a multilingual, multidisciplinary research team that included researchers with backgrounds in public policy, law, economics, public health, and international development, among others. Two researchers independently assessed laws within a standardized coding framework that was developed with feedback from international experts on employment discrimination law and civil society leaders for equal rights.

Where countries’ antidiscrimination policies were primarily legislated at the subnational level, all jurisdictions for that country were assessed and the law with the minimum level of protection was captured. That is, if some states or territories prohibited discrimination based on sexual orientation, but other jurisdictions did not and there was no national level prohibition of discrimination, the country was coded as not prohibiting discrimination based on sexual orientation. If all jurisdictions prohibited a specific aspect of SOGIESC-based discrimination, then the country as a whole was coded as prohibiting that aspect of SOGIESC-based discrimination.

To ensure consistency across the coding team, the team met regularly to discuss any challenging cases where there were disagreements about how to code a country or questions about whether the coding framework specifically covered the case in question. In consultation with senior researchers, once a coding decision was reached, the coding framework was updated to reflect the decision. If this framework revision was likely to impact coding decisions that had already been made for other countries, previously coded countries were rechecked to ensure a consistent application of the revised coding framework.

Antidiscrimination legislation, equal opportunity legislation, labor and employment laws, human rights law, and penal and criminal codes were sourced from the International Labor Organization’s NATLEX database; the coding team’s internal legislative repository that contains legislation identified from more than 15 years of building quantitatively comparable measures of laws and policies; and targeted Google searches. To assess progress on laws prohibiting discrimination over time and in line with international commitments to equality, coding was first conducted with all legislation in force as of 1 August 2016, reflecting legislation in place within a year of the start of the Sustainable Development Goals. Because countries vary as to the amount of time between when legislation is passed and when the new provisions can be enforced, a single date is used for comparability denoting when the legislation came into legal force. The data was updated, and all countries were coded in full again with legislation in force as of 31 May 2023 to assess progress at the mid-point of reaching the Sustainable Development Goals’ 2030 targets. Interactive maps and a public use downloadable dataset are available at worldpolicycenter.org.

### Variables

#### Defining SOGIESC Characteristics

We examined prohibitions of discrimination at work for explicit language prohibiting discrimination based on sexual orientation, gender identity or expression, and sex characteristics. Protections from discrimination on the basis of sexual orientation included terms such as “sexual preference,” “sexual identity,” “homosexual orientation,” or “sexual option.” Protections on the basis of gender identity included terms such as “transgender identity,” “transgender person,” “gender reassignment,” “gender rationale,” or “transsexual.” Protections on the basis of gender expression included terms such as “gender-related appearance or mannerisms.” Protections on the basis of sex characteristics included terms such as “sex characteristics,” “sexual characteristics,” “intersex characteristics,” “intersex status,” or “person with intersex variations.”

#### Defining Other Characteristics

To compare SOGIESC protections with the level of protection for other characteristics, we examined prohibitions of discrimination at work based on sex and/or gender, race and/or ethnicity, and disability status. Prohibitions of discrimination based on sex and/or gender included terms such as “gender” or “sex” or specific protections for “female” or “women” employees (Heymann et al., 2023a, b). Prohibitions of discrimination based on race and/or ethnicity included references to “color,” “clan,” “ethnic origin,” or “ethnic groups” (Heymann et al., 2023a, b). Prohibitions of discrimination based on disability status included general references to disabilities (“handicap,” “impaired,” or “special needs”), as well as specific mentions of mental, intellectual, sensory, or physical disabilities (Heymann et al., 2022).

#### Prohibitions of Discrimination in Aspects of Work

For each protected characteristic, we assessed explicit prohibitions of discrimination across five aspects of an employment relationship: hiring, compensation, training, promotions and/or demotions, and terminations. Protections relevant to hiring include protections specifically extended to job seekers, candidate selection, access to employment, or when filling a vacancy. Protections relevant to compensation include protections specifically extended to guarantees of equal pay, wage, remuneration, salary, or compensation for equal work, or equal pay for work of equal value. Protections relevant to promotions and demotions include protections specifically extended to career advancement and promotion and protections specifically extended against discipline or demotion. Protections relevant to terminations include protections specifically extended to guarantees of job security, job stability, continuance of employment, and protections against wrongful dismissal when discriminatory behavior is also prohibited.

For each aspect, we assessed the strength of protection against SOGIESC-based discrimination. We classified countries as having a “specific prohibition” if legislation either (1) explicitly addressed SOGIESC-based discrimination in that aspect of work (“discrimination based on sexual orientation in hiring is prohibited”) or (2) broadly prohibited SOGIESC-based discrimination at work (“there shall be no discrimination at work based on gender identity”) and guaranteed equality in the specific area (“no one shall be discriminated against in hiring decisions”).

#### Prohibitions of Workplace Harassment

We separately assessed whether laws prohibited both discriminatory harassment based on SOGIESC and whether legislation that prohibited sexual harassment explicitly extended to include harassment based on sexual orientation, same-sex sexual harassment, or defined sexual harassment as a form of discrimination while also prohibiting discrimination based on sexual orientation.

#### Legal Exceptions on the Basis of Religion to Prohibitions of Discrimination

We assessed the degree to which the law explicitly permitted exceptions to prohibitions of SOGIESC-based discrimination at work on the basis of religious beliefs and practices. While freedom of religion is an essential human right recognized under international law, as embodied in the Universal Declaration of Human Rights and the International Covenant on Civil and Political Rights, the selfsame law makes clear that freedom in religiously motivated practices, like all human rights, cannot and should not be used to justify discrimination or other violations of “the fundamental rights and freedoms of others” (International Covenant on Civil and Political Rights, 1966). Seeking to understand when and how the law invokes religious belief and practice in its regulation of discrimination is critical for assessing national compliance with this international consensus.

#### Measures to Support Effective Implementation and Enforcement of Prohibitions

We separately analyzed three aspects of laws that might affect implementation and enforcement. First, we examined whether prohibitions of discrimination included indirect discrimination, which would protect against seemingly neutral practices or criteria that have disparate impacts across SOGIESC characteristics. Second, we assessed whether legislation prohibited retaliation when SOGIESC-based discrimination is reported. We examined both the types of retaliatory action prohibited and whether there was coverage for all employees participating in investigations. Third, we analyzed whether employers were required to take steps to prevent SOGIESC-based discrimination.

#### Legal Gaps that Undermine Prohibitions of Discrimination

Finally, to better understand legal gaps that can perpetuate SOGIESC-based discrimination at work, using data from the International Lesbian, Gay, Bisexual, Trans, and Intersex Association, we assessed other laws that shape individual’s decisions to disclose SOGIESC characteristics and societal norms towards LGBTQI + people: laws criminalizing same-sex sexual behaviors and the legal status of same-sex marriage.

### Analysis

We analyzed the frequency of prohibitions of discrimination based on sexual orientation, gender identity, gender expression, and sex characteristics. We also assessed differences in protections between regions as classified by the World Bank using Pearson’s Chi-squared tests.^1^ Analyses were performed using StataMP 14, and graphs were constructed using R.

## Results

### At Least Some Prohibition of SOGIESC-Based Workplace Discrimination

**Fig. 1:**
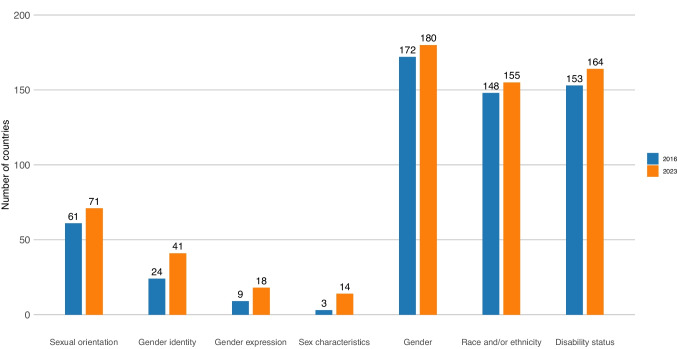
Number of countries prohibiting SOGIESC-based discrimination at work compared to other characteristics in 2016 and 2023

Globally, only 71 countries explicitly prohibited at least some form of workplace discrimination based on sexual orientation (Fig. 1). Far fewer prohibited discrimination based on gender identity (41 countries), gender expression (18 countries), or sex characteristics (14 countries). Three countries (Argentina, India, and Pakistan) prohibited at least some form of workplace discrimination based on gender identity, gender expression, and/or sex characteristics, but did not specifically address equality based on sexual orientation. Prohibitions of SOGIESC-based discrimination at work were far lower than for other protected characteristics, such as sex and/or gender (180 countries), race and/or ethnicity (155 countries), or disability status (164 countries).

There has been an increase in prohibitions of SOGIESC-based discrimination since the start of the Sustainable Development Goals. Between 2016 and 2023, 10 countries passed legislation prohibiting discrimination based on sexual orientation. Four of the countries that passed legislation prohibiting discrimination based on sexual orientation were in sub-Saharan Africa (Angola, Equatorial Guinea, Sao Tome and Principe, and Sierra Leone), three in the Americas (Barbados, Chile, and Honduras), two in East Asia and the Pacific (Mongolia and Tuvalu), and another one in Europe and Central Asia (Iceland). While countries in Europe and Central Asia were most likely to prohibit workplace discrimination based on sexual orientation in both 2016 and 2023 (*p* < 0.01 compared to all regions) (Table 1), only one country in the region has recently passed new laws (Iceland). No countries removed laws prohibiting SOGIESC-based discrimination.

**Table 1:** Prevalence of prohibitions of SOGIESC-based discrimination at work by characteristic, region, and year

	Europe and Central Asia		Americas		East Asia and Pacific		Middle East and North Africa		South Asia		Sub-Saharan Africa	
	2016	2023	2016	2023	2016	2023	2016	2023	2016	2023	2016	2023
Sexual orientation	72%	74%	29% ***	37% ***	17% ***	23% ***	5% ***	5% ***	0% ***	0% ***	15% ***	23% ***
Gender identity	40%	55%	0% ***	17% ***	7% ***	10% ***	0% ***	0% ***	0% ***	25%	2% ***	2% ***
Gender expression	13%	25%	0% **	3% **	7%	10%	0%**	0% **	0%	13%	0% **	0% ***
Sex characteristics	4%	21%	0%	0% ***	3%	3%**	0%**	0%**	0%	25%	0%	0% ***

Chi-squared statistics were calculated separately for the proportion of countries in each region compared to Europe and Central Asia

*** indicates *p* < 0.01. ** indicates* p* < 0.05. * indicates *p* < 0.10

More than a third of countries (17 of 41 countries) with laws prohibiting discrimination based on gender identity passed this legislation between 2016 and 2023. Eight countries that passed these laws were in Europe and Central Asia (Andorra, Bosnia-Herzegovina, Denmark, Iceland, Moldova, the Netherlands, North Macedonia, and Spain), six were in the Americas (Argentina, Canada, Chile, Cuba, Honduras, and Suriname), two were in South Asia (India and Pakistan), and one was in East Asia and Pacific (Mongolia). An additional 9 countries did so for gender expression (Argentina, Andorra, Belgium, Denmark, Iceland, Mongolia, the Netherlands, Pakistan, and Spain). The majority of countries that passed laws prohibiting discrimination based on sex characteristics did so between 2016 and 2023. The 11 countries that passed these provisions were in Europe and Central Asia (Albania, Belgium, Denmark, Greece, Iceland, the Netherlands, Montenegro, Serbia, and Spain) or South Asia (India and Pakistan).

### Prohibitions of Discrimination in Aspects of Work

**Fig. 2:**
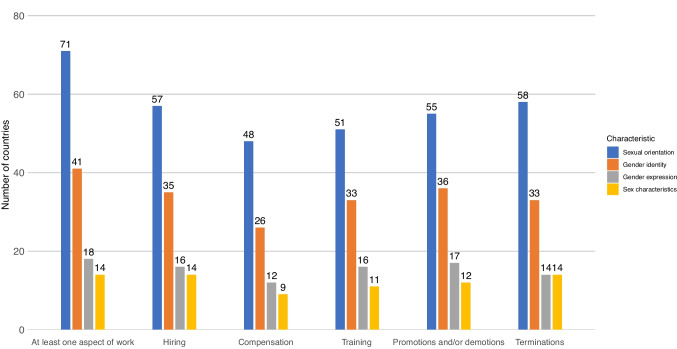
Number of countries prohibiting SOGIESC-based discrimination at work by aspect of work

The most frequently protected aspects of work were hiring, terminations, and promotions and/or demotions. The least commonly protected aspect was compensation discrimination on the basis of sexual orientation (only 48 countries), gender identity (26 countries), gender expression (12 countries), and sex characteristics (9 countries) (Fig. 2).

### Prohibitions of Workplace Harassment

**Fig. 3:**
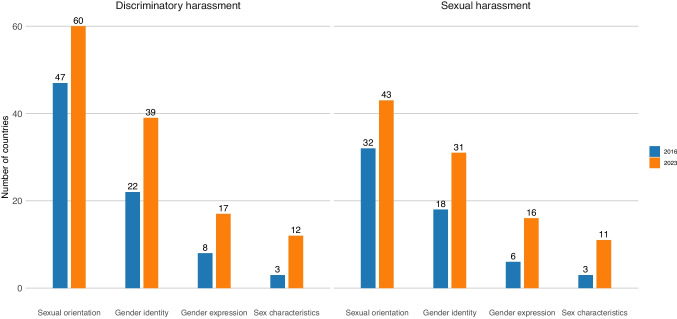
Prohibitions of SOGIESC-based discriminatory and sexual harassment in 2016 and 2023

As of 2023, only 60 countries globally prohibited discriminatory harassment based on sexual orientation, while 39 did so based on gender identity, 17 did so based on gender expression, and only 12 did so based on sex characteristics (Fig. 3). Even fewer countries explicitly prohibited sexual harassment tied to SOGIESC characteristics: just 43 countries explicitly extended prohibitions of workplace sexual harassment to cover harassment based on sexual orientation or same-sex sexual harassment. However, four countries that did not otherwise prohibit workplace discrimination based on sexual orientation did ensure prohibitions of same-sex sexual harassment or sexual harassment based on sexual orientation (Azerbaijan, Bhutan, Peru, and Uruguay).

Prohibitions of harassment have increased over time. Between 2016 and 2023, 13 countries passed laws prohibiting discriminatory harassment based on sexual orientation, 17 did so based on gender identity, 9 did so based on gender expression, and 9 did so based on sex characteristics. While nearly three-quarters of countries in Europe and Central Asia prohibited discriminatory harassment based on sexual orientation, less than a third did so in the Americas and even fewer did so in all other regions (*p* < 0.01 for Europe and Central Asia compared to every other region). Similar disparities across regions were seen for sexual harassment and harassment based on gender identity, gender expression, and sex characteristics. One country that did not otherwise prohibit workplace discrimination based on sexual orientation did ensure prohibitions of discriminatory harassment based on sexual orientation (Bolivia) and two did so for gender identity (Bolivia and Ecuador).

### Legal Exceptions on the Basis of Religion to Prohibitions of Discrimination

Of the 74 countries that prohibited at least some aspect of SOGIESC-based workplace discrimination, the vast majority (60 countries) had no explicit exceptions that allowed for religion to limit prohibitions of SOGIESC-based discrimination. Six countries (Czech Republic, Fiji, Finland, Georgia, Malta, and the UK) more narrowly allowed for exceptions to antidiscrimination provisions for religious practice and/or for leadership positions, employment, or membership in a religious community or order. However, four countries (Bosnia-Herzegovina, Germany, Hungary, and Lithuania) allowed for exceptions for organizations if claimed on the basis of the organization’s ethos or religious convictions. Another four countries (Australia, Barbados, Ireland, and New Zealand) had religious exceptions to workplace antidiscrimination provisions that explicitly extended to cover faith-based institutions that included educational or medical facilities. For example, the Ireland Employment Equality Act allowed exemptions to prohibitions of workplace discrimination for religious, educational, or medical institutions under the direction or control of a body established for religious purposes, while in New Zealand, religious exemptions applied to private school teachers, social workers, counselors, and domestic workers in private households.

### Legal Gaps that Undermine Prohibitions of Discrimination

In addition to these explicit legal exceptions, we also found instances where other factors in a country’s legal environment may potentially impact workers’ ability to exercise an extant prohibition of sexual orientation-based workplace discrimination. Same-sex sexual activities were criminalized in 6 of the 71 countries that offer at least some prohibition of workplace discrimination based on sexual orientation (Kiribati, Liberia, Samoa, Sierra Leone, St. Lucia, and Tuvalu), seemingly putting lesbian, gay, bisexual, and queer workers at risk of legal incrimination in addition to discrimination. In Kiribati, for instance, despite Sects. 153, 154, and 155 of its Penal Code outlawing same-sex sexual activity, its Employment and Industrial Relations Code expressly prohibits discrimination against employees based on sexual orientation. Same-sex marriage was also not legal in 41 of those countries, potentially leading LGBTQI + workers with same-sex partners to face unequal barriers in accessing the same workplace benefits and family-related leaves accorded to those in legally recognized different-sex relationships. Three countries that extended antidiscrimination protections relevant to transgender and gender diverse workers also exclusively did so on the basis of gender reassignment or sex change (Bulgaria, Luxembourg, and the UK). The specificity of this terminology potentially limits transgender and gender diverse workers’ ability to access legal protections on the basis of their gender identity or gender expression without the provable involvement of a process to change their sex.

### Measures to Support Effective Implementation and Enforcement of Prohibitions

**Fig. 4:**
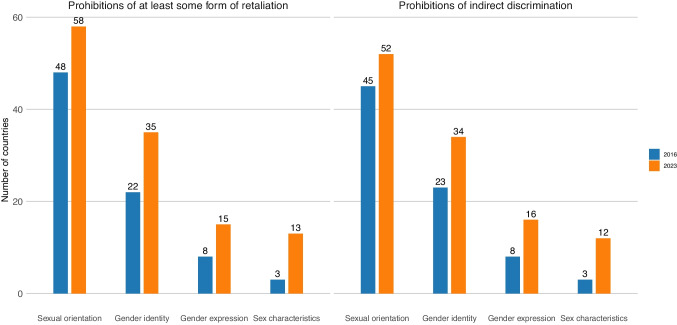
Measures to support implementation and enforcement of laws prohibiting SOGIESC-based discrimination at work in 2016 and 2023

While the majority of countries that prohibited SOGIESC-based discrimination at work also took steps that matter for their laws’ implementation and enforcement, important gaps remained (Fig. 4). A failure to ban all forms of discriminatory practices creates one such gap. For instance, workplace discrimination can emerge indirectly through apparently neutral policies or activities, such as only extending workplace benefits to opposite-sex spouses or partners, requiring adherence to a gender binary-conforming dress code, not permitting changes to records of employees’ name and gender, or arranging non-essential events in LGBTQI + hostile environments. Only 52 of the 71 countries that prohibited at least some form of discrimination based on sexual orientation explicitly ensured that this covered indirect discrimination, whereas 34 of 41 countries did so for gender identity, 16 of 18 countries did so for gender expression, and 12 of 14 did so for sex characteristics. While all countries in Europe & Central Asia and East Asia & Pacific regions that prohibited discrimination based on sexual orientation specifically covered indirect discrimination, a third of sub-Saharan African countries and 15% of countries in the Americas did so (*p* < 0.01 for each region compared to Europe and Central Asia).

Most countries prohibited at least some form of retaliation for reporting SOGIESC-based discrimination at work. Of the 71 countries that prohibited discrimination based on sexual orientation, 58 also prohibited retaliation. However, in one of these countries, protections were limited to retaliatory dismissal and one country only covered disciplinary sanctions. Thirty-five of the 41 countries that prohibited discrimination based on gender identity, 15 of the 18 countries that prohibited discrimination based on gender expression, and 13 of the 14 that prohibited discrimination based on sex characteristics also prohibited retaliation. All countries that prohibited retaliatory action for reporting discrimination based on gender identity, gender expression, or sex characteristics specifically covered any adverse action. Fewer countries prohibited retaliation against workers who participated in workplace investigations of discrimination based on sexual orientation (45 countries), gender identity (26 countries), gender expression (12 countries), or sex characteristics (7 countries). In nearly every region, at least one country failed to ensure that workers who report discrimination based on sexual orientation are protected from retaliation.^2^ These gaps were most prevalent in sub-Saharan Africa (45% of countries that prohibit discrimination fail to prohibit retaliation) and the Americas (38%) (*p* < 0.01 for each of these regions compared to Europe and Central Asia).

Only a minority of countries prohibiting discrimination at work based on sexual orientation also required employers to take specific steps to prevent this discrimination (23 of 71 countries), such as raising awareness of discrimination laws or providing training, and an additional 6 countries made it a general requirement but did not specify measures. While 18 of 41 countries made it a requirement for employers to prevent discrimination based on gender identity, and 10 of 18 countries did so for gender expression, only 4 did so for sex characteristics. Europe and Central Asia is the only region where the majority of countries (62%) prohibiting discrimination based on sexual orientation also make it the employer’s responsibility to prevent this discrimination. No countries in the Middle East and North Africa did so.

Since 2016, 7 countries have passed laws prohibiting indirect workplace discrimination based on sexual orientation, 12 have done so for gender identity, 8 for gender expression, and 9 for sex characteristics. Similarly, 10 countries have passed legislation prohibiting at least some form of retaliatory action for reporting sexual orientation discrimination, 14 have done so for gender identity, 7 for gender expression, and 10 for sex characteristics.

## Discussion

Comprehensive antidiscrimination labor laws represent the first step in dismantling the inequitable barriers LGBTQI + people face in accessing work, maintaining access to safe and supportive workplace environments, and guaranteeing access for all to meaningful socioeconomic opportunity. The data demonstrate sizable progress in the enactment of these protections. From 2016 to 2023 alone, more than a third of all national prohibitions of workplace discrimination in at least one aspect of work on the basis of gender identity became law, as did half of all such prohibitions on the basis of gender expression and 79% on the basis of sex characteristics or intersex status. These represent notable gains in legal protections for gender diverse and intersex workers. Progress in protections across the SOGIESC spectrum has also been comparatively steady, with all groups matching or exceeding the number of countries that have added prohibitions of discrimination at work on the basis of race and/or ethnicity in the same 2016 to 2023 time period.

However, progress has not been distributed equally around the world. While all regions have at least one country with prohibitions of SOGIESC-based workplace discrimination, large gaps remain in the East Asia, North Africa and Middle East, and sub-Saharan Africa regions. Future research should examine the dynamics promoting and inhibiting policy progress in these countries. The six countries with prohibitions of SOGIESC-based workplace discrimination despite laws criminalizing same-sex activity could serve as valuable case studies regarding advocacy and passage of antidiscrimination policies in LGBTQI + -polarized and low public acceptance environments. Decriminalization in these countries is essential to non-discrimination becoming meaningful.

Conversely, the data highlights countries whose policy pathways have led to the legal protection of LGBTQI + rights except in regards to national employment antidiscrimination law. Four of the 36 countries that have legalized or plan to legalize same-sex marriage (Argentina, Brazil, the USA, and Uruguay), for instance, had no explicit national prohibition on workplace discrimination on the basis of sexual orientation. These countries instead leave the enactment of these protections to subnational governments, creating inequities in national coverage. Alternatively, protections are encoded in potentially mutable case law. Moving to universally prohibit discrimination at work without unpredictable variations in physical or legal geography is an important priority that should be readily achievable.

Even in countries with national discrimination prohibitions, there remains room for improvement in prohibition specificity. In regards to a vital aspect of work like pay, for instance, only 48 countries explicitly prohibited discrimination in compensation on the basis of sexual orientation, only 26 did so on the basis of gender identity, only 12 did so on the basis of gender expression, and only 9 did so on the basis of sex characteristics. Despite LGBTQI + workers’ disproportionate rates of workplace sexual harassment (Brassel et al., 2019), sexual harassment remains one of the prohibitions least commonly extended to explicitly cover SOGIESC-based discrimination. The need for more expansive protections in legal enforcement mechanisms to ensure policy efficacy is also evident. In 13 countries, laws prohibited discrimination based on sexual orientation, but lacked anti-retaliation provisions to protect those who report discrimination from termination.

Closing these critical legal gaps is a feasible step that all countries should take to benefit human rights, strengthen economies, and lay the foundation for norm change. In addition, countries should address other potential obstacles to the exercise of discrimination prohibitions, such as barriers to accessing the judicial system. Future research in this policy area should consider access to legal aid in discrimination cases and means for workers to address discrimination through non-judicial avenues and complaints procedures.

Regardless of the strength of protections, though, they are negated when they land in the lacunae of explicit exceptions to the law. Of the 71 countries that prohibited at least some aspect of SOGIESC-based discrimination at work, four provided exemptions for faith-based institutions. Importantly, the overwhelming majority of nations with protections from discrimination recognize, as does international law, that religious belief is not a justification for discriminatory behavior. However, the exceptions permitted by these four countries not only undermined LGBTQI + people’s legal protection from workplace discrimination, but their equitable access to other fundamental human rights in education and health as well.

This study created a new quantitative database measuring laws and policies prohibiting SOGIESC-based discrimination at work. This type of data has been used by civil society and policymakers to push for legislative change in other policy spheres by highlighting where change has been feasible in other similar countries (Heymann et al., 2023a, b; Raub et al., 2022). This database provides transparent, actionable monitoring and accountability by showing whether countries are taking a first step towards fulfilling their international commitments to equality by passing laws that prohibit SOGIESC-based discrimination. Additionally, it serves as a potential resource for researchers examining the effects of antidiscrimination laws. Previous studies have used quantitative measures of laws and policies to rigorously assess the relationship between laws and policies on health outcomes, economic outcomes, and norms (Flores & Park, 2018; Nyeck et al., 2019; Solazzo et al., 2018). Detailed quantitative measures can also answer questions about whether certain policy features, such as protection from retaliation or requirements for employers to prevent discrimination, are necessary to support effective implementation.

This study has several important limitations. First, while this study provides the first globally comparative examination of progress in workplace antidiscrimination protections based on sexual orientation, gender identity, gender expression, and sex characteristics, globally comparative data is not available on the extent of those protections’ implementation. We are able to begin to address this limitation by capturing aspects of the laws that support implementation. Future work should examine barriers and supports to help LGBTQI + people access justice when their legal rights have been violated and the role of businesses in advancing a more inclusive work environment.

Secondly, judicial interpretations of non-discrimination laws have effectively expanded coverage for LGBTQI + people in some countries. Notably, clauses that prohibit discrimination on the basis of gender have been interpreted by some courts to include sexual orientation, leveraging a broader understanding of sex discrimination to offer protections. For example, in the USA, the Supreme Court’s decision in *Bostock v. Clayton County* (2020) clarified that Title VII of the Civil Rights Act of 1964’s prohibition against employment discrimination “because of…sex” extends to sexual orientation and gender identity. Such case law developments provide an important avenue for adapting existing legal frameworks to recognize and protect the rights of LGBTQI + people. However, while gender-based protections are significant, they may not inherently or sufficiently cover the specific needs of the LGBTQI + community. For clarity, gender can be defined in legislation to include SOGIESC grounds. Greece’s Law on the Principle of Equal Treatment of Men and Women in Matters of Employment, for instance, explicitly delineates protections for sexual orientation and gender identity alongside sex. By doing so, such countries create a legislative framework that distinctly and intentionally safeguards LGBTQI + workers, leaving no ambiguity about the intent or extent of its protections. This explicitness in legislation is crucial. It is not enough for countries to rely on gender protections alone to infer protection for LGBTQI + people; specific and overt legal measures are necessary to provide robust and lasting workplace equality.

Similarly, universal prohibitions of discrimination have sometimes been interpreted as protecting the rights of LGBTQI + people. For example, the Supreme Court of India recognized a constitutional right to non-discrimination on the basis of sexual orientation in *Navtej Singh Johar & Others v. Union of India* (2018), and the Supreme Court of Nepal extended its constitution’s right to equality to include all LGBTI people in *Sunil Babu Pant and Others v. Nepal Government and Others* (2007). However, these judicial interpretations are not systematically captured in this study due to the inherent difficulties in monitoring case law, regulations, and other forms of implementation across 193 countries. Protections established through case law also carry the risk of being more susceptible to reversal with changes in political leadership or judicial composition compared to those codified in legislation. The highly polarized recognition of LGBTQI + rights across different countries underscores the importance of specifically enshrining these rights in legislation, ensuring their durability, and providing a clear standard for enforcement.

While this study was able to capture data on the existence of religious exceptions or loopholes in the legislative protections, other exceptions may impact the strength of legislation. Additional countries have legal exceptions to antidiscrimination laws for discrimination undertaken for “genuine,” “valid,” or “justified” reasons, or in defense of “public morals.” Data are not available on how often such language has been used to undermine protections. Further research should examine the risks and impacts of these explicit exceptions in the law in greater depth.

Finally, this data focuses on prohibitions of discrimination for individuals working in the private sector. While research from other domains suggest that laws covering the formal economy may have spillover benefits or egalitarian normative value for wider society (Chai et al., 2022; Omidakhsh et al., 2020), legal protection and access to justice may be limited for workers in the informal economy. Moreover, this is only one of the needed protections from discrimination for LGBTQI + people. Non-discrimination in all areas, including the provision of goods and services, housing, education, and healthcare, among others, is essential. Additionally, non-discrimination cannot be effectively realized until systemic discrimination is eliminated across policy domains, from the decriminalization of same-sex sexual activity to the full legal recognition of intersex people and non-binary gender.

That said, addressing discrimination in employment can still have a great systemic impact, given its vast implications for the economic and personal health, well-being, and livelihoods of LGBTQI + adults, their biological and chosen families, and their communities in every country. By adopting the Sustainable Development Goals and/or ratifying the International Covenant on Economic, Social, and Cultural Rights, every country has already committed to ensuring the economic inclusion and equal rights of LGBTQI + workers. Yet significant gaps remain in most countries’ legal frameworks. Banning SOGIESC-based discrimination at work in every country is but one step toward enabling all LGBTQI + people to live to their fullest potential—but, in the interest of ensuring equal rights for every worker, step forward we must.

## Data Availability

A public use dataset will be made freely available upon publication at worldpolicycenter.org.

## Footnotes

1 As a member of the European Union, we chose to classify Malta as a part of Europe and Central Asia for the purposes of interpreting results. All other countries retained their World Bank regional classifications.

2 Only one country in the Middle East and North Africa prohibits sexual orientation-based discrimination. It also prohibits retaliation.

## Code Availability

Code is available from the corresponding author upon request.

## References

[CR1] Aksoy, C. G., Carpenter, C. S., Frank, J., & Huffman, M. L. (2019). Gay glass ceilings: Sexual orientation and workplace authority in the UK. *Journal of Economic Behavior and Organization,**159*, 167–180. 10.1016/j.jebo.2019.01.013

[CR2] Badgett, M. V. L., Waaldijk, K., & Rodgers, Y. van der M. (2019). The relationship between LGBT inclusion and economic development: Macro-level evidence. *World Development*, *120*, 1–14. 10.1016/j.worlddev.2019.03.011

[CR3] Barron, L. G., & Hebl, M. (2013). The force of law: The effects of sexual orientation antidiscrimination legislation on interpersonal discrimination in employment. *Psychology, Public Policy, and Law,**19*(2), 191–205. 10.1037/a0028350

[CR4] Bostock v. Clayton County, 590 U.S. 644 (2020) (U.S. Supreme Court). Decided June 15, 2020.

[CR5] Brown, C., Contreras, D., & Schmidt, L. (2019). Sexual orientation and labor force participation: Findings from Chile and Uruguay. *Feminist Economics,**25*(2), 90–115. 10.1080/13545701.2018.1554905

[CR6] Bryson, A. (2017). Pay equity after the Equality Act 2010: Does sexual orientation still matter? *Work, Employment and Society,**31*(3), 483–500. 10.1177/0950017016664678

[CR7] Burn, I. (2020). The relationship between prejudice and wage penalties for gay men in the United States. *ILR Review,**73*(3), 650–675. 10.1177/0019793919864891

[CR8] Burn, I. (2018). Not all laws are created equal: Legal differences in state non-discrimination laws and the impact of LGBT employment protections. In *Journal of Labor Research* (Vol. 39, Issue 4). Journal of Labor Research. 10.1007/s12122-018-9272-0

[CR9] Chai, Y., Ríos-Salas, V., Stek, P., & Heymann, J. (2022). Does enhancing paid maternity leave policy help promote gender equality? Evidence from 31 low- and middle-income countries. *Gender Issues,**39*(3), 335–367. 10.1007/s12147-021-09293-4 35875727 10.1007/s12147-021-09293-4 PMC9300538

[CR10] Collins, W. J. (2003). The labor market impact of state-level anti-discrimination laws, 1940–1960. *ILR Review,**56*(2), 244–272. 10.1177/001979390305600203

[CR11] Costa, A. B., Brum, G. M., Zoltowski, A. P. C., Dutra-Thomé, L., Lobato, M. I. R., Nardi, H. C., & Koller, S. H. (2020). Experiences of discrimination and inclusion of brazilian transgender people in the labor market. *Revista Psicologia: Organizações e Trabalho*, *20*(2), 1040–1046. 10.17652/rpot/2020.2.18204

[CR12] Cramer, R. J., Kaniuka, A. R., Yada, F. N., Diaz-Garelli, F., Hill, R. M., Bowling, J., Macchia, J. M., & Tucker, R. P. (2022). An analysis of suicidal thoughts and behaviors among transgender and gender diverse adults. *Social Psychiatry and Psychiatric Epidemiology,**57*(1), 195–205. 10.1007/s00127-021-02115-8 34106286 10.1007/s00127-021-02115-8

[CR13] Day, N. E., & Schoenrade, P. (2000). The relationship among reported disclosure of sexual orientation, anti-discrimination policies, top management support and work attitudes of gay and lesbian employees. *Personnel Review,**29*(3), 346–363. 10.1108/00483480010324706

[CR14] Delhommer, S. (2020). Effect of state and local sexual orientation anti-discrimination laws on labor market differentials. 10.2139/ssrn.3625193

[CR15] Dilmaghani, M., & Robinson, M. (2022). The blue of the rainbow: Queerness and hiring discrimination in blue-collar occupations. *Review of Social Economy*. 10.1080/00346764.2022.2030491

[CR16] Donohue, J. J., & Heckman, J. J. (1991). *Continuous versus episodic change: The impact of civil rights policy on the economic status of blacks*. National Bureau of Economic Research Cambridge, Mass., USA.

[CR17] Drydakis, N. (2011). Women’s sexual orientation and labor market outcomes in Greece. *Feminist Economics,**17*(1), 89–117. 10.1080/13545701.2010.541858

[CR18] Drydakis, N. (2015). Sexual orientation discrimination in the United Kingdom’s labour market: A field experiment. *Human Relations,**68*(11), 1769–1796. 10.1177/0018726715569855

[CR19] Drydakis, N. (2021a). Sexual orientation discrimination in the labor market against gay men. *Review of Economics of the Household*. 10.1007/s11150-021-09581-8

[CR20] Drydakis, N. (2022). Sexual orientation and earnings: A meta-analysis 2012–2020. *Journal of Population Economics,**35*(2), 409–440. 10.1007/s00148-021-00862-1

[CR21] Drydakis, N., & Zimmermann, K. F. (2020). *Sexual orientation, gender identity and labour market outcomes: New Patterns and insights* (627; GLO Discussion Paper). Global Labor Organization (GLO). http://hdl.handle.net/10419/223005

[CR22] Drydakis, N. (2021b). *The economics of being LGBT. A review: 2015–2020* (14845; IZA Discussion Papers). IZA - Institute of Labor Economics. http://hdl.handle.net/10419/246076

[CR23] European Union Agency for Fundamental Rights. (2015). *Being trans in the EU – Comparative analysis of the EU LGBT survey data – Summary*. https://fra.europa.eu/sites/default/files/fra-2015-being-trans-eu-comparative-summary_en.pdf

[CR24] Everett, B. G., Limburg, A., McKetta, S., & Hatzenbuehler, M. L. (2022). State-level regulations regarding the protection of sexual minorities and birth outcomes: Results from a population-based cohort study. *Psychosomatic Medicine, Publish Ah.*10.1097/PSY.0000000000001092 10.1097/PSY.0000000000001092 PMC9271587 35471976

[CR25] Flage, A. (2020). Discrimination against gays and lesbians in hiring decisions: A meta-analysis. *International Journal of Manpower*, *41*. 10.1108/IJM-08-2018-0239

[CR26] Flores, A. R., & Park, A. (2018). *Examining the relationship between social acceptance of LGBT people and legal inclusion of sexual minorities* (Issue March, p. 29). The Williams Institute.

[CR27] Flores, A. R., & Barclay, S. (2016). Backlash, consensus, legitimacy, or polarization: The effect of same-sex marriage policy on mass attitudes. *Political Research Quarterly,**69*(1), 43–56. 10.1177/1065912915621175

[CR28] Granberg, M., Andersson, P. A., & Ahmed, A. (2020). Hiring discrimination against transgender people: Evidence from a field experiment. *Labour Economics,**65*(May), 101860. 10.1016/j.labeco.2020.101860

[CR29] Gunderson, M. (1989). Male-female wage differentials and policy responses. *Journal of Economic Literature,**27*(1), 46–72.

[CR30] Herman, A., Brown, J. L., & Haas, T. N. (2019). *Suicide thoughts and attempts among transgender adults: Findings from the 2015 U.S. transgender survey* (pp. 1–35). The Williams Institute. https://escholarship.org/uc/item/1812g3hm

[CR31] Heymann, J., Wong, E., & Waisath, W. (2022). A comparative overview of disability-related employment laws and policies in 193 countries. *Journal of Disability Policy Studies,**33*(1), 25–34. 10.1177/10442073211006396

[CR32] Heymann, J., Sprague, A., & Raub, A. (2023a). *Equality within our lifetimes*. University of California Press. 10.1525/luminos.147

[CR33] Heymann, J., Varvaro-Toney, S., Raub, A., Kabir, F., & Sprague, A. (2023b). Race, ethnicity, and discrimination at work: A new analysis of legal protections and gaps in all 193 UN countries. *Equality, Diversity and Inclusion: An International Journal,**42*(9), 16–34. 10.1108/EDI-01-2022-0027

[CR34] ILGA World, Mendos, L. R., Botha, K., Lelis, R. C., de la Peña, E. L., Savelev, I., & Tan, D. (2020). State sponsored homophobia 2020: Global legislation overview update. ILGA.

[CR35] United Nations (General Assembly) (1966). “International Covenant on Civil and Political Rights.” *Treaty Series, 999*, 171.

[CR36] James, S. E., Herman, J. L., Rankin, S., Keisling, M., Mottet, L., & Anafi, M. (2016). *The report of the 2015 U.S. transgender survey* (p. 302). National Center for Healthcare Equality. http://www.transequality.org/sites/default/files/docs/USTS-Full-Report-FINAL.PDF

[CR37] Kenny, C., & Patel, D. (2017). Norms and reform: Legalizing homosexuality improves attitudes. *Center for Global Development Working Paper*, *465*.

[CR38] Laurent, T., & Mihoubi, F. (2017). Sexual orientation, unemployment and participation: Are gays less employable than straights? *Journal of Labor Research,**38*(1), 1–44. 10.1007/s12122-016-9237-0

[CR39] Martell, M. E. (2013). Do ENDAs end discrimination for behaviorally gay men? *Journal of Labor Research,**34*(2), 147–169. 10.1007/s12122-012-9154-9

[CR40] Martell, M. E. (2014). How ENDAs extend the workweek: Legal protection and the labor supply of behaviorally gay men. *Contemporary Economic Policy,**32*(3), 560–577. 10.1111/coep.12035

[CR41] Moya, M., & Moya-Garófano, A. (2020). Discrimination, work stress, and psychological well-being in LGBTI workers in Spain. *Psychosocial Intervention,**29*(2), 93–101. 10.5093/pi2020a5

[CR42] Nyeck, S. N., Shepherd, D., Sehoole, J., Ngcobozi, L., & Conron, K. J. (2019). *The economic cost of LGBT stigma and discrimination in South Africa*. Williams Institute on Sexual Orientation and Gender Identity Law and Public Policy. https://williamsinstitute.law.ucla.edu/wp-content/uploads/Impact-LGBT-Discrimination-South-Africa-Dec-2019.pdf

[CR43] Office of the United Nations High Commissioner for Human Rights. (2019). *Born Free and Equal: Sexual Orientation, Gender Identity and Sex Characteristics in International Human Rights Law (2nd ed.)*. https://www.ohchr.org/sites/default/files/Documents/Publications/Born_Free_and_Equal_WEB.pdf

[CR44] Omidakhsh, N., Sprague, A., & Heymann, J. (2020). Dismantling restrictive gender norms: Can better designed paternal leave policies help? *Analyses of Social Issues and Public Policy,**20*(1), 382–396. 10.1111/asap.12205

[CR45] Navtej Singh Johar & Others v. Union of India, through the Secretary Ministry of Law and Justice, 2018 INSC 746 (Supreme Court of India). Decided September 6, 2018.

[CR46] Ozeren, E. (2014). Sexual orientation discrimination in the workplace: A systematic review of literature. *Procedia - Social and Behavioral Sciences,**109*, 1203–1215. 10.1016/j.sbspro.2013.12.613

[CR47] Pérez Álvarez, A., Correa Montoya, G., Castañeda Castro, W., & Plata Chacón, E. (2013). *Raros... Y oficios: Diversidad sexual y mundo laboral: Discriminación y exclusión*.

[CR48] Pichler, S., Varma, A., & Bruce, T. (2010). Heterosexism in employment decisions: The role of job misfit. *Journal of Applied Social Psychology,**40*(10), 2527–2555. 10.1111/j.1559-1816.2010.00669.x

[CR49] Pichler, S., Blazovich, J. L., Cook, K. A., Huston, J. M., & Strawser, W. R. (2018). Do LGBT-supportive corporate policies enhance firm performance? *Human Resource Management,**57*(1), 263–278. 10.1002/hrm.21831

[CR50] Poushter, J., & Kent, N. O. (2020). *The global divide on homosexuality persists*. Pew Research Center. https://www.pewresearch.org/global/2020/06/25/global-divide-on-homosexuality-persists/

[CR51] Preston, A., Birch, E., & Timming, A. R. (2020). Sexual orientation and wage discrimination: Evidence from Australia. *International Journal of Manpower,**41*(6), 629–648. 10.1108/IJM-08-2018-0279

[CR52] Raub, A., Cassola, A., Latz, I., & Heymann, J. (2016). Protections of equal rights across sexual orientation and gender identity: An analysis of 193 national constitutions. *Yale Journal of Law and Feminism,**28*(1), 149–170.

[CR53] Raub, A., Sprague, A., Waisath, W., Nandi, A., Atabay, E., Vincent, I., Moreno, G., Earle, A., Perry, N., & Heymann, J. (2022). Utilizing a comparative policy resource from the WORLD Policy Analysis Center covering constitutional rights, laws, and policies across 193 countries for outcome analysis, monitoring, and accountability. *Journal of Comparative Policy Analysis: Research and Practice,**24*(4), 313–328. 10.1080/13876988.2021.1894073

[CR54] Riggle, E. D. B., Rostosky, S. S., & Horne, S. (2010). Does it matter where you live? Nondiscrimination laws and the experiences of LGB residents. *Sexuality Research and Social Policy,**7*(3), 168–175. 10.1007/s13178-010-0016-z

[CR55] Sears, B., Mallory, C., Flores, A. R., & Conron, K. J. (2021). *LGBT people’s experiences of workplace discrimination and harassment* (Issue September). The Williams Institute. https://williamsinstitute.law.ucla.edu/publications/lgbt-workplace-discrimination/

[CR56] Solazzo, A., Brown, T. N., & Gorman, B. K. (2018). State-level climate, anti-discrimination law, and sexual minority health status: An ecological study. *Social Science and Medicine*, *196*(September 2017), 158–165. 10.1016/j.socscimed.2017.11.033 10.1016/j.socscimed.2017.11.033 29190536

[CR57] Suárez, M. I., Marquez-Velarde, G., Glass, C., & Miller, G. H. (2020). Cis-normativity at work: Exploring discrimination against US trans workers. *Gender in Management*. 10.1108/GM-06-2020-0201

[CR58] Suen, Y. T., Chan, R. C. H., & Badgett, M. V. L. (2021). The experiences of sexual and gender minorities in employment: Evidence from a large-scale survey of lesbian, gay, bisexual, transgender and intersex people in China. *The China Quarterly,**245*, 142–164. 10.1017/S0305741020000429

[CR59] Sunil Babu Pant & Others v Nepal Government & Others, Writ Petition No. 917 of 2007 (Supreme Court of Nepal). Decided December 21, 2007.

[CR60] Tilcsik, A. (2011). Pride and prejudice: Employment discrimination against openly gay men in the United States. *American Journal of Sociology,**117*(2), 586–626. 10.1086/661653 10.1086/661653 22268247

[CR61] TUC. (2019). *Sexual harassment of LGBT people in the workplace* (pp. 1–36). https://www.tuc.org.uk/sites/default/files/LGBT_Sexual_Harassment_Report_0.pdf

[CR62] UN Committee on Economic, Social and Cultural Rights. (2016). *General comment no. 22 (2016) on the right to sexual and reproductive health (article 12 of the International Covenant on Economic, Social and Cultural Rights)*. https://digitallibrary.un.org/record/832961

[CR63] Valfort, M.-A. (2017). *LGBTI in OECD countries: A review* (198; OECD Social, Employment and Migration Working Papers). OECD Publishing. 10.1787/d5d49711-en

[CR64] Vu, T. V. (2022). Linking LGBT inclusion and national innovative capacity. *Social Indicators Research,**159*(1), 191–214. 10.1007/s11205-021-02743-2

[CR65] Waaldijk, C. (2009). *Legal recognition of homosexual orientation in the countries of the world. A chronological overview with footnotes*. https://hdl.handle.net/1887/14543

[CR66] Wang, J., & Gunderson, M. (2019). Can pay gaps between gay men and lesbians shed light on male–female pay gaps? *International Journal of Manpower,**40*(2), 178–189. 10.1108/IJM-11-2017-0298

[CR67] Weichselbaumer, D. (2022). Discrimination due to sexual orientation. In *Handbook of Labor, Human Resources and Population Economics* (pp. 1–27). Springer International Publishing. 10.1007/978-3-319-57365-6_301-1

[CR68] Wong, E., Jou, J., Raub, A., & Heymann, J. (2020). Comparing the availability of paid parental leave for same-sex and different-sex couples in 34 OECD countries. *Journal of Social Policy,**49*(3), 525–545. 10.1017/S0047279419000643

[CR69] Wright, G. (2015). The regional economic impact of the Civil Rights Act of 1964. *Boston University Law Review,**95*(3), 759–780.

